# Avenanthramide supplementation reduces eccentric exercise-induced inflammation in young men and women

**DOI:** 10.1186/s12970-020-00368-3

**Published:** 2020-07-25

**Authors:** Tianou Zhang, Tong Zhao, Yuzi Zhang, Tao Liu, Gilles Gagnon, Jacqueline Ebrahim, Jodee Johnson, Yi-Fang Chu, Li Li Ji

**Affiliations:** 1 Laboratory of Exercise and Sports Nutrition (LESN), Department of Kinesiology, The University of Texas at San Antonio, San Antonio, TX 78249 USA; 2 Laboratory of Physiological Hygiene and Exercise Science (LPHES), School of Kinesiology, University of Minnesota-Twin Cities, Minneapolis, MN 55455 USA; 3 Ceapro Inc., Edmonton, AB T6E 6W2 Canada; 4 PepsiCo R&D Nutrition, Barrington, IL 60010 USA

**Keywords:** Avenanthramide, Downhill running, Inflammation, Chemokines, Cytokines

## Abstract

**Background:**

Avenanthramides (AVA) are a group of di-phenolic acids found only in oats and have shown antioxidant and anti-inflammatory effects in vitro and in vivo. Eccentric muscle contraction is intimately involved in rigorous exercise that activates systemic and local inflammatory responses. The objective of the study is to evaluate whether chronic AVA supplementation could attenuate peripheral inflammatory and immunological markers in human subjects in response to an acute bout of downhill running (DR).

**Methods:**

Eleven male and thirteen female subjects voluntarily participated in this double-blinded, randomized controlled study and were randomly divided into AVA-supplemented (AVA) or control (C) groups. All subjects conducted a DR protocol at − 10% grade with an intensity equivalent to 75% of their maximal heart rate. Blood samples were collected at rest and various time points (0-72 h) after DR (PRE). After an 8-week washout period, participants received two cookies daily containing either 206 mg/kg (AVA) or 0 mg/kg (C) AVA for 8 weeks. Following the oat supplementation regimen, the DR and blood sampling protocols were repeated (POST). Plasma inflammatory and immunological markers were measured using Multiplex immunoassay and muscle soreness was evaluated with pain rating scale.

**Results:**

DR increased plasma creatine kinase (CK) activity (*P* < 0.01) during PRE, but the response was reduced at 24 and 48 h during POST vs. PRE regardless of AVA status (*P* < 0.05). Neutrophil respiratory burst (NRB) levels were elevated at 4 and 24 h (*P* < 0.05) during PRE but were significantly decreased at 0–48 h during POST vs. PRE (*P* < 0.05 or 0.01). Granulocyte-colony stimulating factor (G-CSF), the neutrophil stimulating cytokine, was also increased in response to DR but showed lower levels in AVA compared to C during POST vs. PRE (*P* < 0.05). Plasma interleukin-6 (IL-6) content showed an increase at 0 and 4 h during PRE and 0 h during POST (*P* < 0.01), whereas during POST there was a trend toward a lower IL-6 level in AVA vs. C (*P* = 0.082). Plasma levels of anti-inflammatory agent interleukin-1 receptor antagonist (IL-1Ra) showed an increase at 4 h during PRE, and was significantly elevated in AVA vs. C during POST. Both soluble vascular cell adhesion molecule-1 (sVCAM-1) and monocyte chemoattractant protein-1 (MCP-1) contents increased at 0 and 24 h post DR during PRE as well as POST sessions, however, sVCAM-1 content was lower in AVA vs. C during POST (*P* < 0.05) and MCP-1 levels were below resting level at 24, 48 and 72 h during POST (*P* < 0.05). DR increased muscle pain at all post-DR time points (*P* < 0.01), but the pain level was alleviated by oat supplementation at 48 and 72 h during POST regardless of AVA treatment (*P* < 0.05).

**Conclusions:**

Oat AVA supplementation reduced circulatory inflammatory cytokines and inhibited expression of chemokines and cell adhesion molecules induced by DR.

**Trial registration:**

ClinicalTrials.gov identifier: NCT02584946. Registered 23 October 2015.

## Introduction

Eccentric exercise-induced muscle damage is a major physio-pathological problem associated with unaccustomed exercise and sports. Downhill running (DR) as one form of eccentric exercise lengthens lower-extremity muscle groups during contraction, leading to macro and micro muscle injury, such as reduced excitation-contraction coupling, sarcomere disruption, torn myofibers, and microstructural damage [1–4]. Damaged muscle fibers are known to release pro-inflammatory cytokines such as interleukin (IL)-1β and tumor necrosis alpha (TNF-α), which stimulate the expression of various cell adhesion molecules (CAMs), such as intercellular adhesion molecules (ICAMs) and vascular adhesion molecules (VCAMs) on the surface of endothelial cells. Furthermore, these interactions induce chemoattractants (e.g. MCP-1) and pro-inflammatory cytokines (e.g., IL-6, IL-8) release [5]. Adhesion molecules expressed on the surface of endothelial cells can attract phagocytic cells like monocytes and neutrophils to migrate to the injury site [6]. Monocytes are differentiated into M1 macrophage which promotes inflammation, while neutrophils are involved in the neutrophil respiratory burst (NRB) that produce reactive oxygen species (ROS) catalyzed by NADPH oxidase [7–9]. These spacial and temporal events take place during 0 to 24 h after the cessation of eccentric exercise. It is noteworthy that although the above cytokines and myokines participate in and promote muscle inflammatory responses (0 to 48 h) after injury and can lead to reduced muscle metabolic and contractile function, they also play important roles in muscular regeneration and remodeling during the recovery process [9].

Due to the critical role which inflammation plays in eccentric exercise-induced muscle damage, research has long been focused on containing and reducing inflammatory responses using various pharmaceutical, physical and nutritional means [10]. Among these treatment strategies, avenanthramides (AVA), a group of diphenolic compounds found only in oats, have demonstrated potent anti-inflammatory functions in vitro and in vivo [11–16]. As the name implies, the structure of AVA includes an anthranilate derivative and a phenylpropanoid derivative linked by an amide (pseudo peptide) bond [17]. Previous studies have shown that AVA are bioavailable to rats [18] and human [19, 20]. Importantly, the anti-inflammatory effect AVA demonstrated in various studies is linked to its ability to inhibit NF-κB activity and the expression of pro-inflammatory cytokines and chemotactins [15, 16, 21–23]. For example, 8 weeks of dietary AVA supplementation was shown to effectively reduce DR-induced muscle damage (marked by creatine kinase, CK, release), neutrophil respiratory burst (NRB) and plasma inflammatory cytokine levels in college-age women [14]. Furthermore, pain sensation resulting from lengthening muscle contraction during DR was attenuated among the subjects. These findings suggest that AVA may be used as a potential nutritional supplement in coping with muscle and systemic inflammation after an exercise stress.

Despite the efficacy AVA has demonstrated in vitro and in vivo, the precise mechanism by which it inhibits immunoreactive cells to regulate chemokines and cell adhesion molecules is largely unclear. Furthermore, all the previous clinical studies on AVA were conducted in female subjects, and this gender-specific feature has shed some doubts on AVA’s general utility as a dietary supplement. Thus, the purpose of the study is to evaluate the effects of chronic AVA supplementation on peripheral blood inflammatory markers induced by DR in both male and female subjects. We hypothesized that: (1) DR could cause significant muscle damage and associated increases in plasma pro-inflammatory cytokines and chemokines in human subjects; (2) AVA supplementation would decrease DR-induced muscle damage, neutrophil ROS generation, and inflammatory cytokine and chemokine levels in blood circulation; and (3) AVA supplementation would reduce subjects’ pain sensation during and after DR.

## Methods

### Subjects

Eleven male and thirteen female non-obese subjects (Age: 23.0 ± 1.2 years, BMI: 22.3 ± 0.8 kg/m^2^, HR_max_: 191.3 ± 2.3 bpm) were recruited from the Twin Cities community to participate in this double-blinded, randomized controlled trial (ClinicalTrials.gov identifier: NCT02584946). All participants were instructed to sign informed consent approved by Institutional Review Board at University of Minnesota (UMN) before being enrolled into the study. Participants were asked to take the Health History and Gastrointestinal Tolerability Questionnaires to ensure that they met selection criteria. The Exclusion Criteria included gastrointestinal malabsorption, clinically significant disorders of circulatory, respiratory, digestive, urinary and nervous systems and related treatment medications, major trauma or surgery in the past 3 months, cancer in the prior 2 years, smoking, drinking alcohol > 5 drinks/week, allergic to oat products, nonsteroidal anti-inflammatory drugs (NSAIDS> 800 mg ibuprofen/week), and using nutraceuticals and vitamin supplementation. Women who are pregnant or lactating were also excluded. Over the counter or prescription medication consumption within the past 4 weeks were recorded. The following people who had moderate-intensity cardiorespiratory exercise ≥150 min/week (≥30 min/d on ≥5 d/week), vigorous-intensity cardiorespiratory exercise ≥75 min/week (≥20 min/d on ≥3 d/week), or a combination of moderate- and vigorous-intensity exercise to achieve a total energy expenditure of ≥500–1000 MET/min·wk. were not permitted to participate in the study [24].

### Dietary supplementation

Participants were randomly assigned to two dietary groups, consuming oat cookies containing AVA (*n* = 12) or minimal AVA (C, *n* = 12) daily for 8 weeks. The cookies AVA group received contained 206 mg/kg AVA, whereas the cookies C group received had non-detectable AVA. Each cookie was made of 30 g oat flour and baked at 212 °F for 15 min to avoid excessive AVA degradation. Final AVA contents in the AVA and C cookie was 10.3 mg/cookie (20.6 mg/day) and 0 mg/cookie (0 mg/day), respectively.

### Downhill running protocol

**Fig. 1:**
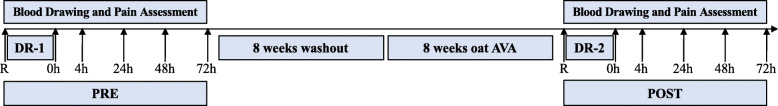
Study Design

A maximal exercise test (VO_2max_ test) was performed experiment began to estimate maximum heart rate (HR_max_) and calculate target heart rate for the DR test. After an overnight fast, the participants reported to the Laboratory of Physiological Hygiene and Exercise Science to perform a DR protocol. After warm-up on the treadmill for 5 min at 0% grade, the subjects started 4 bouts of DR with 15 min each at − 10% grade. The treadmill speed was manually adjusted at 5-min intervals to keep the participant’s heart rate at 75% of HR_max_. DR bouts were separated by 5 min of rest. Blood samples were taken at rest, immediately after DR (0 h), and at 4, 24, 48 and 72 h post DR. Rated perceived exertion (RPE) score on 6–20 scale (Borg scale) was evaluated in the last minute of each of the 15 min DR, and at each time point of during the post-DR time period. This DR protocol performed prior to dietary supplementation regimen (PRE) was followed by an 8-week washout period, and repeated after the 8-week dietary supplementation as described before (POST). The DR protocols and testing conditions were also kept the same during both sessions, such as the subjects’ relative heart rate (%HR_max_), room temperature, humidity and ventilation. All sessions were monitored by laboratory personnel who were trained in First Aid and exercise physiology. The entire experimental design of the study is depicted in Fig. 1.

### Blood collection and biomarker measurement

Mixed venous blood was drawn from the antecubital vein into one EDTA-coated vacutainer tube and one heparin sodium-coated vacutainer tube (10 ml each). 100 μl whole blood was transferred from EDTA-coated vacutainer tube to a 5 ml polystyrene tube for NRB test on flow cytometer. The heparin sodium-coated tube was immediately centrifuged at 3000 x g for 15 min at 4 °C to obtain plasma. Plasma was removed by aspiration and frozen at − 80 °C.

### Muscle damage marker and blood ROS production

Plasma creatine kinase (CK) activity was measured as a marker of muscle fiber damage with a colorimetric method using a microplate reader (BioAssay Systems). Briefly, 10 μl plasma samples were added into separate wells in a 96-well plate, and 100 μl reconstituted reagents (10 μl substrate solution, 100 μl assay buffer and 1 μl enzyme mix) were added. Samples were incubated at 37 °C for 20 min and the plate was read at OD340nm at 20th min and 40th min.

Neutrophil Respiratory Burst (NRB) was measured with a neutrophil respiratory burst assay kit (Cayman) and data were collected by BD Accuri C6 flow cytometer. Briefly, 100 μl whole blood was added to a polypropylene tube containing 10 μl 10X dihydrorhodamine (DHR)-123. After incubation at 37 °C for 15 min, 25 μl 5X phorbol myristate acetate (PMA) was added and the assay mixture was incubated at 37 °C for 45 min. After addition of 2 ml red blood cell (RBC) lysis buffer, the tube was incubated at 37 °C for 10 min and centrifuged for 10 min at room temperature at 500×g. Supernatant was discarded and the cell pellet was resuspended in 0.5 ml assay buffer. DHR-123 was converted to a fluorescent compound rhodamine-123 by ROS, and the latter one emitted a green fluorescence (~ 530 nm) similar to fluorescein isothiocyanate (FITC) which was detectable in the FL-1 channel of the flow cytometer.

### Cell adhesion molecules and inflammatory cytokines

Inflammatory cytokine (IL-6), anti-inflammatory cytokine (IL-1Ra), cell adhesion molecule (sVCAM-1), colony stimulating factor (G-CSF) and chemotactic cytokine (MCP-1) were measured with Multiplex assay (R&D System). In general, 12.5 μl human plasma sample was added to a mixture of color-coded beads pre-coated with antibodies that capture specific analytes. Biotinylated detection antibodies were added and formed an antibody-antigen sandwich specific to the analytes, and phycoerythrin (PE)-conjugated streptavidin were bound to the biotinylated detection antibodies. Polystyrene beads were detected on Bio-Plex 200 System (Bio-Rad), with one laser determining the analyte and the second laser measuring the magnitude of the PE-derived signal in proportion to the amount of analyte bound.

### Pain rating scale

For all visits, ratings of pain and leg muscle soreness were collected using a visual analog scale after instructing participants to squat to an approximate knee angle of 90° and with hands on hips. Participants were prompted to place a vertical line along a 10 cm line segment with the left terminus representing no pain or soreness and the right terminus representing the worst possible pain or soreness. The distance from the left terminus to the vertical line was measured in millimeters and recorded.

### Data analysis

A three-way repeated measure ANOVA was conducted using SPSS (version 22) statistical software. The three main factors are (a) Supplementation: POST vs. PRE; (b) Exercise: timing with respect to DR; and (c) AVA: AVA vs. C. The standard error of estimate of the ANOVA was used to complete the planned comparisons. Significance level was adjusted and set at the quotient of 0.05 divided the number of comparison groups. Interaction effects were also measured between Supplementation, Exercise and AVA.

## Results

### Muscle damage and oxidative stress markers

**Fig. 2:**
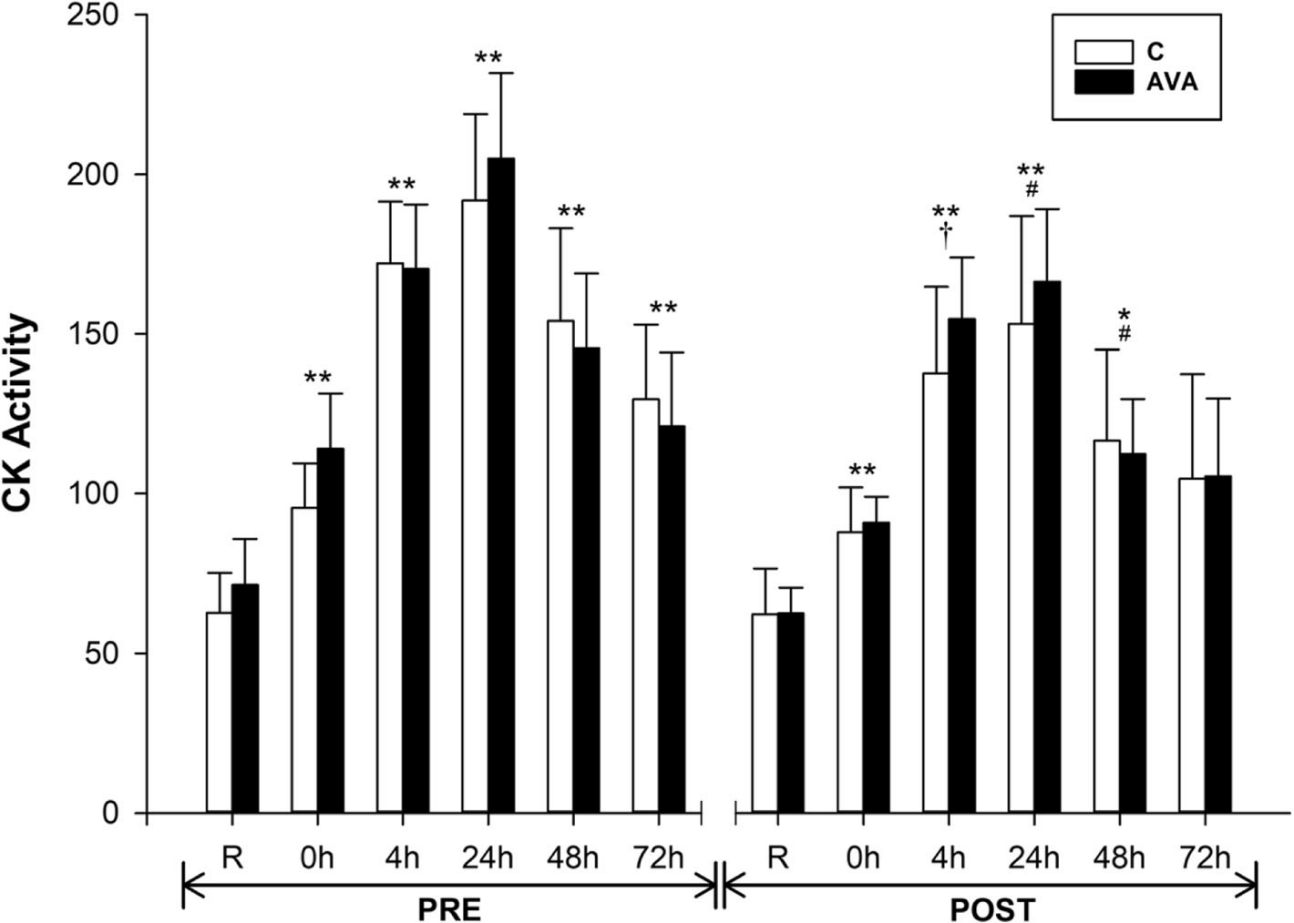
Plasma CK activity. Data are shown as mean ± SEM. Exercise effect: ^*^P < 0.05 or ^**^P < 0.01, 0 h/4 h/24 h/48 h/72 h post-DR vs. Rest. Supplementation effect: ^#^*P* < 0.05 or †*P* = 0.094, POST vs. PRE regardless of time or AVA treatment. Interaction effect: *P* < 0.05, time×supplementation

Plasma CK activity, a well-known marker of muscle membrane damage and leakage, was significantly increased after DR at all time points (*P* < 0.01) during PRE session (Fig. 2). During the POST session, CK activity was also elevated following DR at most time points (*P* < 0.05), but not at 72 h. Overall, CK activity during POST session was significantly decreased compared to PRE session (*P* < 0.05, time×supplementation), while at 24 and 48 h it was 19.5 and 23.6% lower (*P* < 0.05), respectively, than their PRE counterparts. No difference in CK activity was found between subjects receiving AVA or C treatment.

**Fig. 3:**
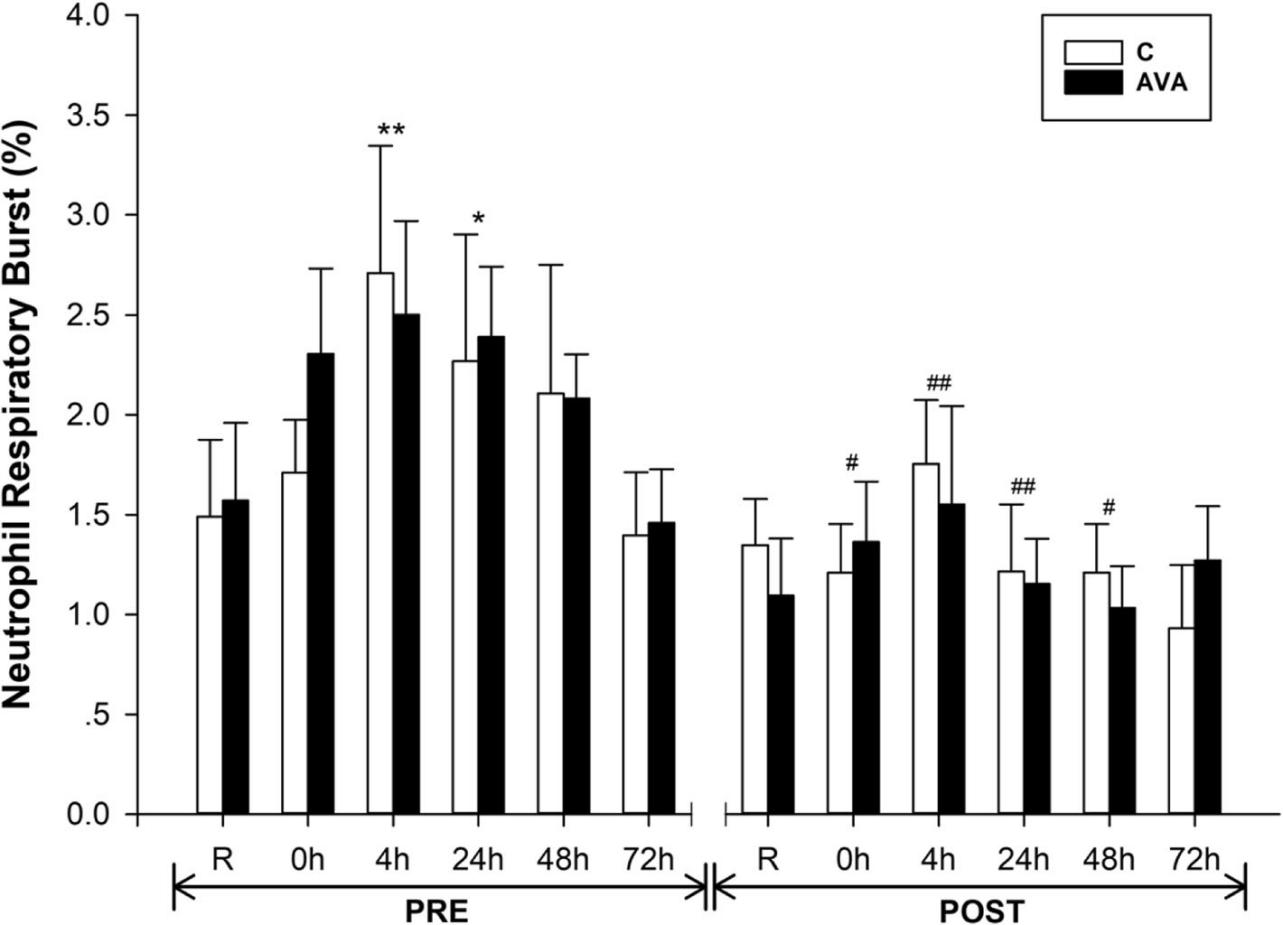
Neutrophil Respiratory Burst (NRB). Data are shown as mean ± SEM. Exercise effect: ^*^P < 0.05 or ^**^P < 0.01, 0 h/4 h/24 h/48 h/72 h post-DR vs. Rest. Supplementation effect: ^#^*P* < 0.05 or ^##^*P* < 0.01, POST vs. PRE regardless of time or AVA treatment. Interaction effect: *P* < 0.05, time×AVA; *P* = 0.060, supplementation × AVA; *P* = 0.059, time×supplementation

NRB was measured in blood samples collected immediately following each visit as a biomarker for ROS generation (Fig. 3). During PRE session, NRB was elevated by 70.2% (*P* < 0.01) at 4 h and 52.2% (*P* < 0.05) at 24 h following DR. These DR-induced changes, however, were not observed during POST session. NRB level was lowered at 0 h (*P* < 0.05), 4 h and 24 h (*P* < 0.01), and 48 h (*P* < 0.05) during POST session compared to the respective values during PRE session. Furthermore, there was a significant interaction effect between time and AVA dose (*P* < 0.05) during POST session, showing AVA-supplemented group had lower NRB percentage than C.

**Fig. 4:**
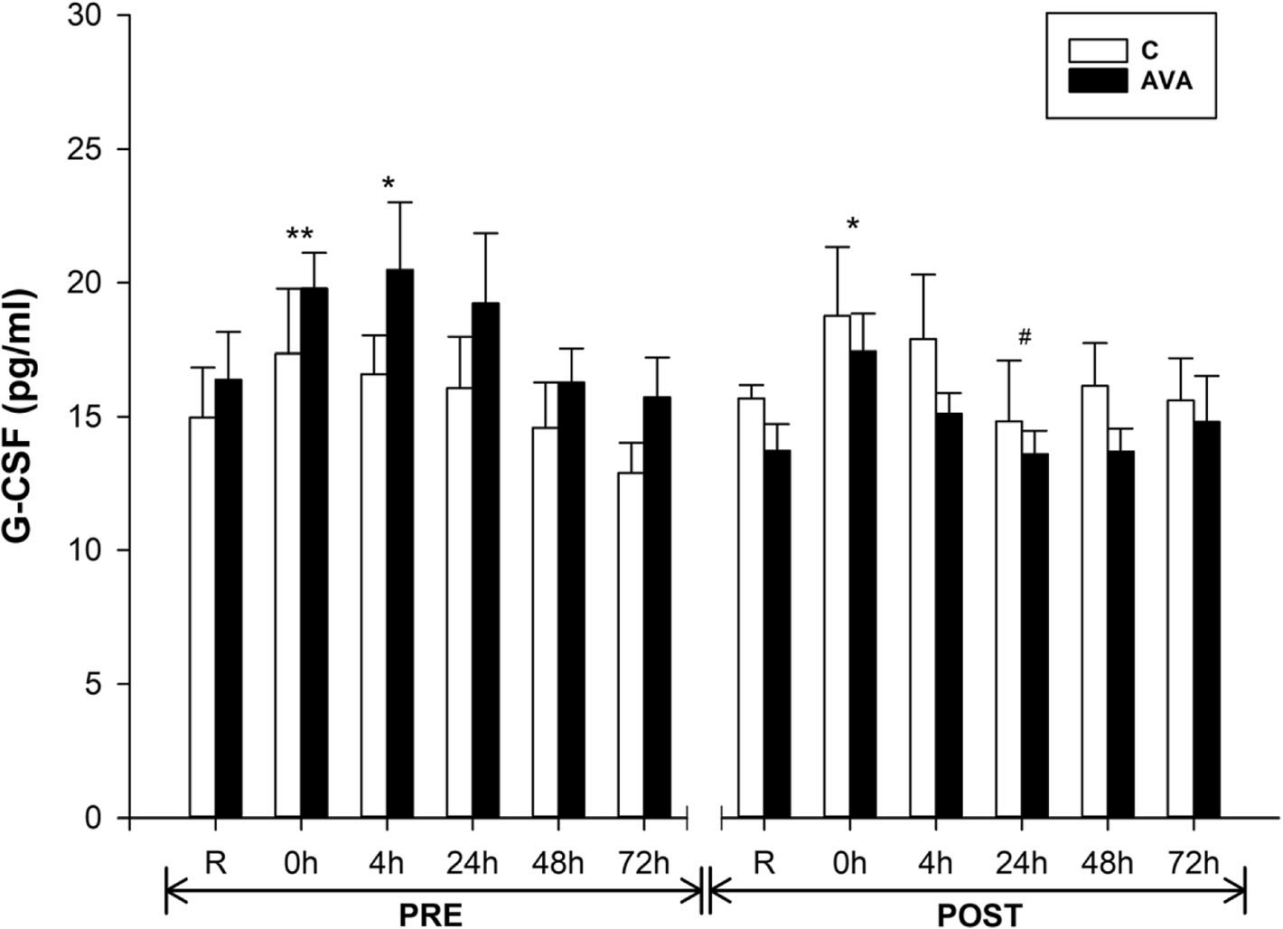
Plasma G-CSF concentrations. Data are shown as mean ± SEM. Exercise effect: ^*^P < 0.05 or ^**^P < 0.01, 0 h/4 h/24 h/48 h/72 h post-DR vs. Rest. Supplementation effect: ^#^*P* = 0.056, POST vs. PRE regardless of time or AVA treatment. Interaction effect: *P* < 0.05, supplementation×AVA

G-CSF stimulates the proliferation of granulocytes and is one of the most important regulators of neutrophil function. Plasma G-CSF concentrations was significantly increased at 0 h (*P* < 0.01) and 4 h (*P* < 0.05) after DR during PRE session, but returned to R values thereafter (Fig. 4). During POST session, however, G-CSF was elevated only at 0 h (*P* < 0.05). Also, G-CSF levels were reduced at 24 h compared to its counterpart value during PRE session (*P* = 0.056). Furthermore, AVA-supplemented participants showed overall lower G-CSF levels than participants receiving C treatment during the POST session, but not during the PRE session (*P* < 0.05, supplementation×AVA).

### Plasma inflammatory and anti-inflammatory cytokines

**Fig. 5:**
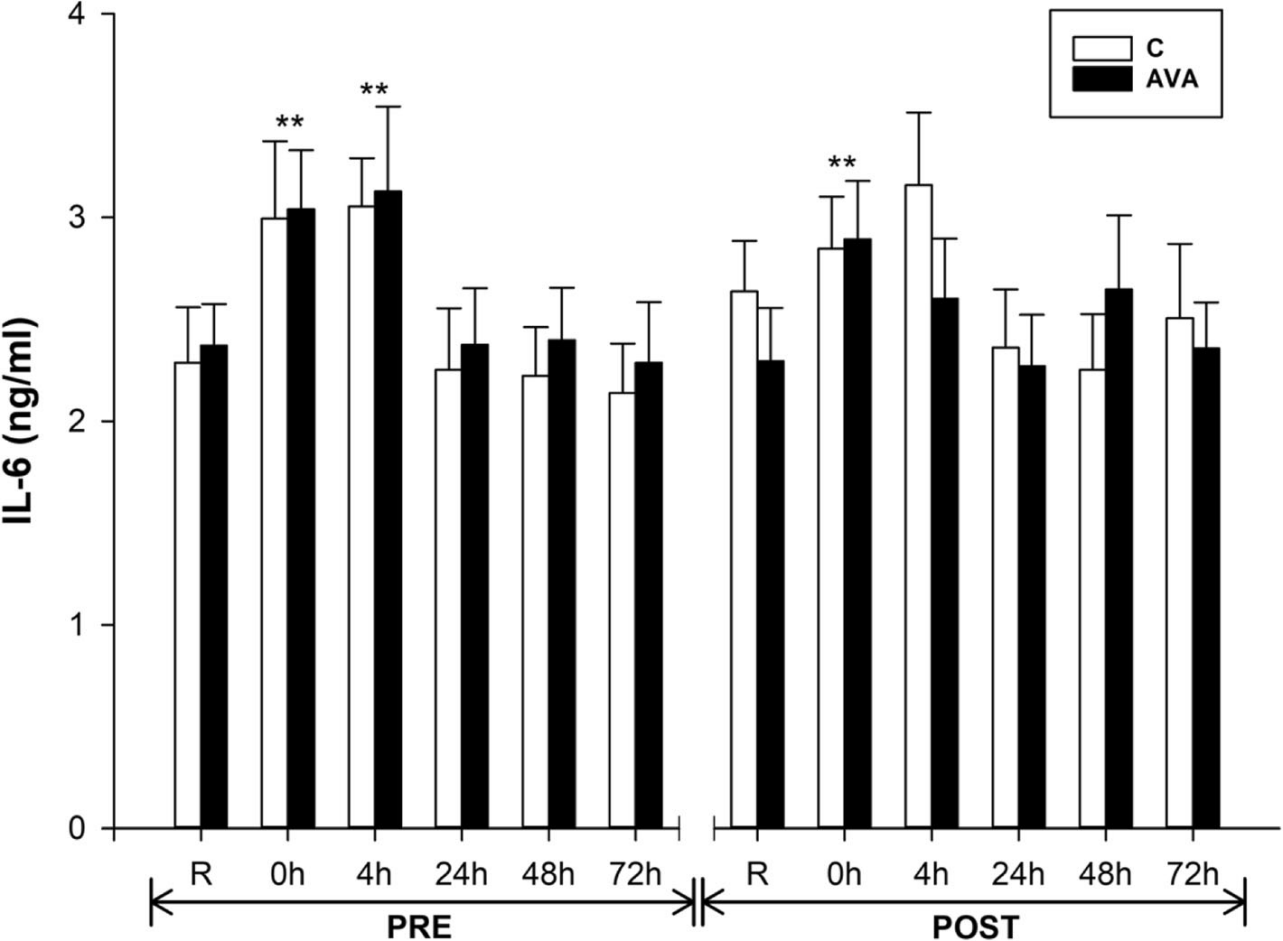
Plasma IL-6 concentrations. Data are shown as mean ± SEM. Exercise effect: ^**^P < 0.01, 0 h/4 h/24 h/48 h/72 h post-DR vs. Rest. Interaction effect: *P* = 0.082, time×AVA

Plasma concentrations of IL-6, an important pro-inflammatory cytokine, was significantly increased at 0 h (*P* < 0.01) and 4 h (*P* < 0.01) after DR during PRE session, whereas it was elevated only at 0 h (*P* < 0.01) during POST session (Fig. 5). In addition, IL-6 levels tended to be lower during POST session in the AVA-supplemented group than the C group (*P* = 0.082, time×AVA).

**Fig. 6:**
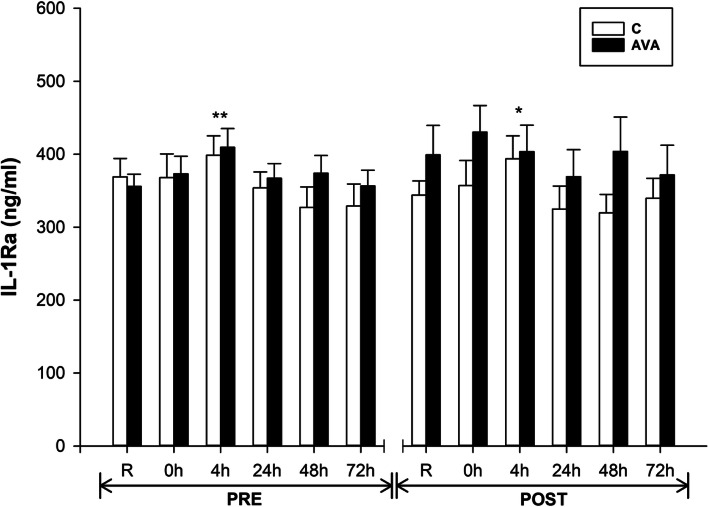
Plasma IL-1Ra concentrations. Data are shown as mean ± SEM. Exercise effect: ^*^P = 0.069 or ^**^P < 0.01, 0 h/4 h/24 h/48 h/72 h post-DR vs. Rest. Interaction effect: *P* < 0.05, time×supplementation×AVA

IL-1Ra is a natural inhibitor of IL-1β and modulate a variety of IL-1 related immune and inflammatory responses. Plasma IL-1Ra concentrations showed a small but significant increase at 4 h during PRE (*P* < 0.01) and a trend toward a higher level at 4 h during POST (*P* = 0.069) (Fig. 6). Also, IL-1Ra levels were significantly higher in AVA group than C group during the POST session (*P* < 0.05, time×supplementation×AVA).

### Cell adhesion molecule and chemokine

**Fig. 7:**
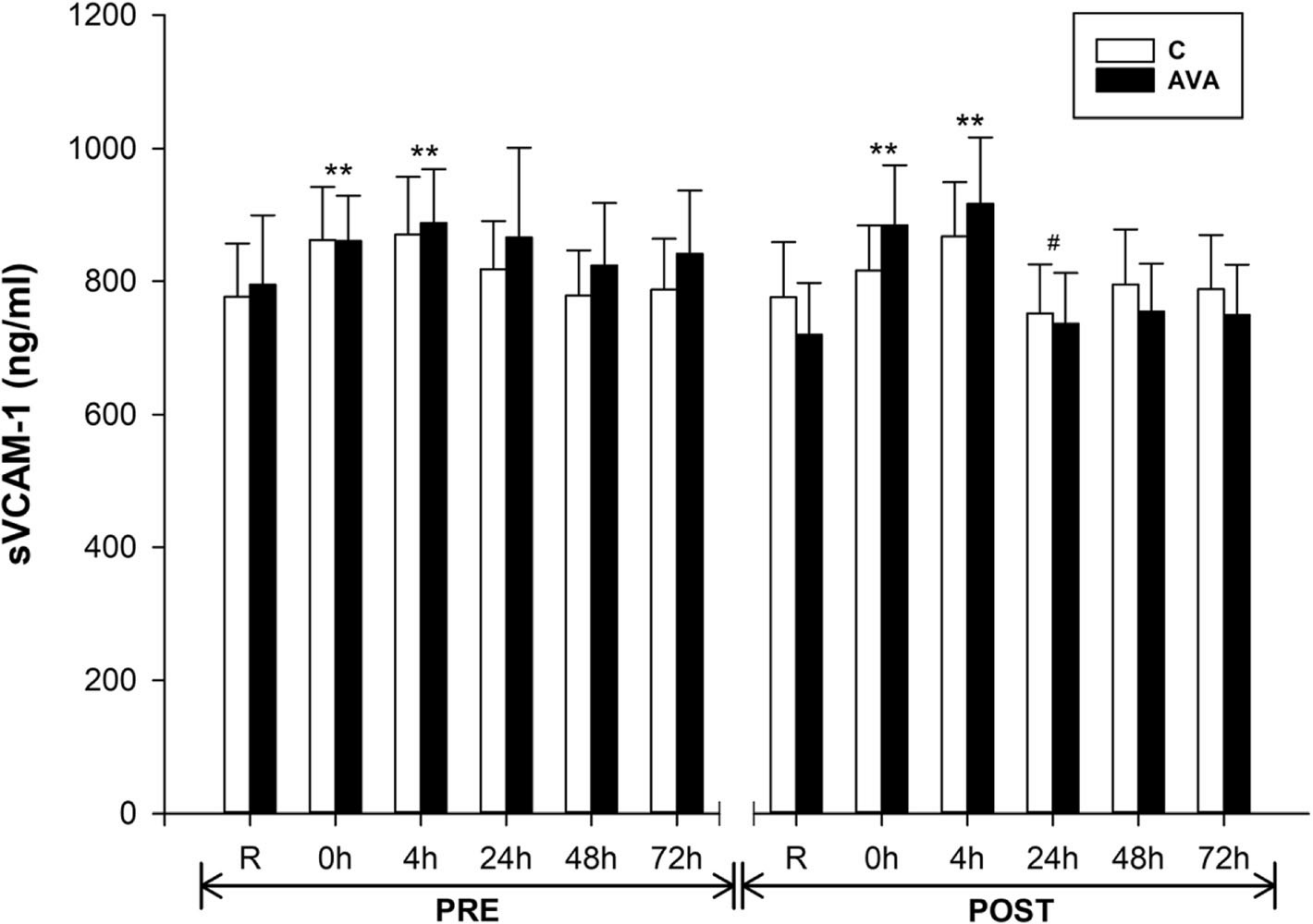
Plasma sVCAM-1 concentrations. Data are shown as mean ± SEM. Exercise effect: ^**^P < 0.01, 0 h/4 h/24 h/48 h/72 h post-DR vs. Rest. Supplementation effect: ^#^*P* = 0.079, POST vs. PRE regardless of time or AVA treatment. Interaction effect: *P* < 0.05, time×AVA

To further test our hypothesis that AVA might inhibit DR-induced chemokine and adhesion molecule expression, we measured plasma sVCAM-1 and MCP-1 concentrations in response to DR (Fig. 7). sVCAM-1 levels were elevated at 0 h (*P* < 0.01) and 4 h (*P* < 0.01) after DR during both PRE and POST sessions. sVCAM-1 showed a tendency toward a lower response to DR at 24 h during POST than PRE (*P* = 0.079). Moreover, during POST, AVA-supplemented group had reduced sVCAM-1 response to DR than C group (*P* < 0.05, time×AVA).

**Fig. 8:**
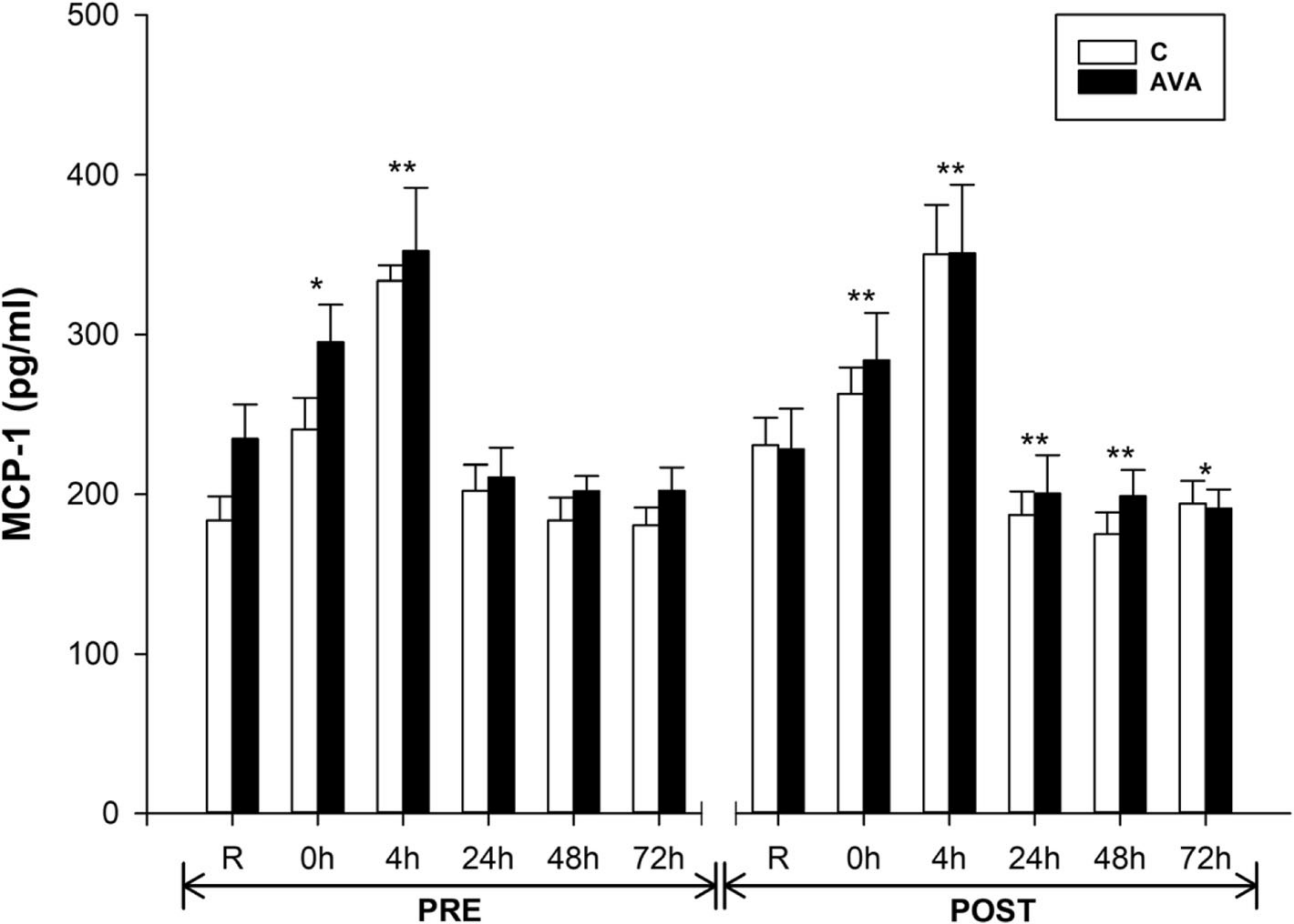
Plasma MCP-1 concentrations. Data are shown as mean ± SEM. Exercise effect: ^*^P < 0.05 or ^**^P < 0.01, 0 h/4 h/24 h/48 h/72 h post-DR vs. Rest. Interaction effect: *P* = 0.064, time×supplementation

Another important chemokine, MCP-1, also clearly showed elevated plasma levels in response to DR at 0 h (*P* < 0.05) and 4 h (*P* < 0.01) during PRE, and at 0 and 4 h (*P* < 0.01) during POST (Fig. 8). However, during PRE, MCP-1 level returned to R level after 24 h, but during POST, it was decreased at 24, 48 (*P* < 0.01) and 72 h (*P* < 0.05). A trend was revealed showing lower MCP-1 levels during POST compared to those during PRE over time (*P* = 0.064, time×supplementation).

### Pain rating scale

**Fig. 9:**
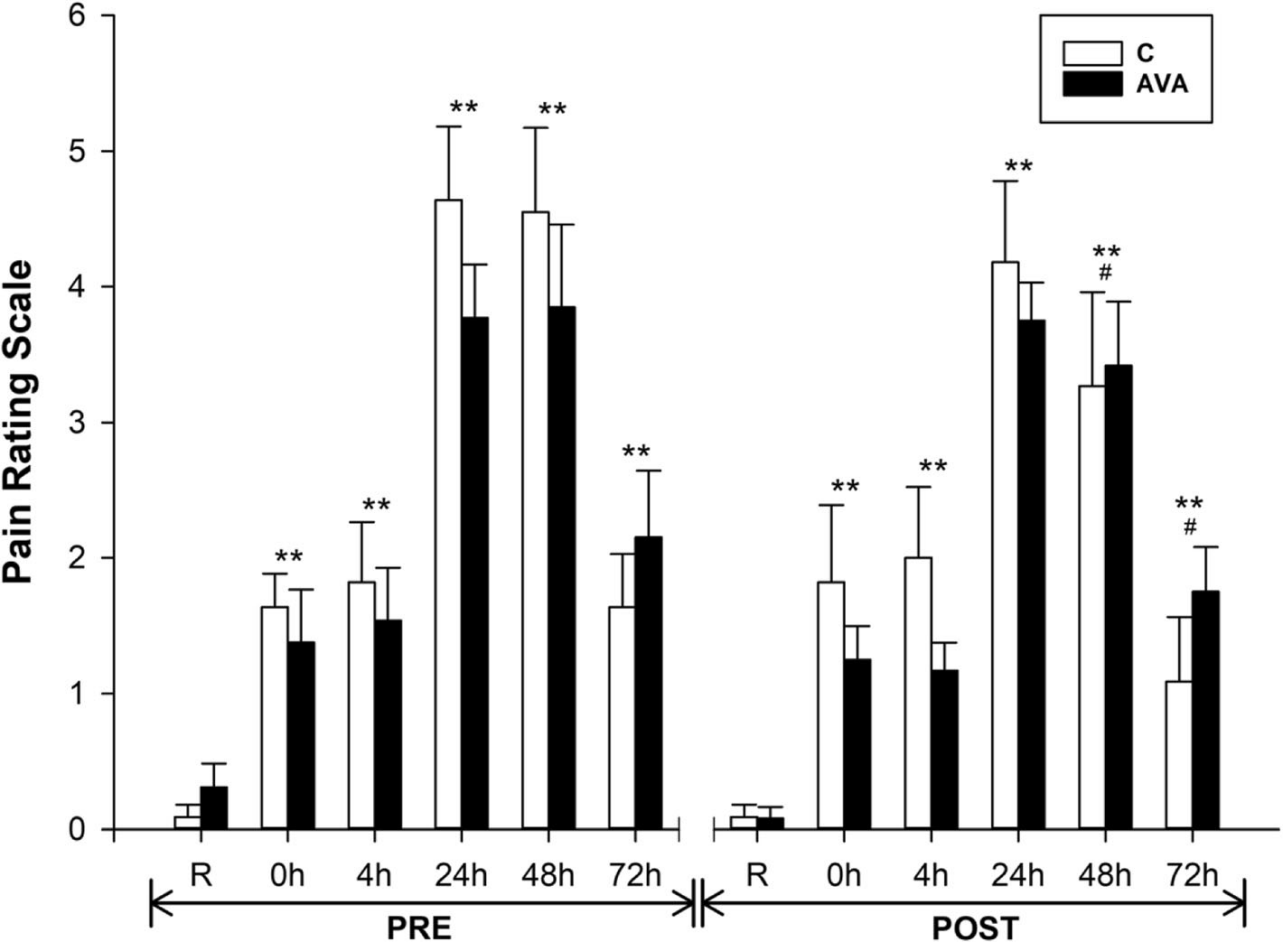
Pain rating scale. Data are shown as mean ± SEM. Exercise effect: ^**^P < 0.01, 0 h/4 h/24 h/48 h/72 h post-DR vs. Rest. Supplementation effect: ^#^*P* < 0.05, POST vs. PRE regardless of time or AVA treatment. Interaction effect: *P* < 0.05, time×supplementation

DR significantly increased the muscle pain ratings among participants, shown during both PRE and POST sessions (Fig. 9). Pain reached the peak at 24 and 48 h (*P* < 0.01), but sustained through 72 h (*P* < 0.01). However, pain levels were significantly lower during POST compared to PRE (*P* < 0.05, time×supplementation). Furthermore, pain ratings showed significant lower levels at 48 and 72 h during POST than their PRE counterparts (*P* < 0.05).

## Discussion

With the increasing demand for physical activity to promote a healthier lifestyle among the general public, maintaining proper antioxidant defense during exercise and sports is an important issue for muscle health [25, 26]. Eccentric contraction (EC), also known as lengthening contraction, is an integral element in high-intensity physical activity and sports [27, 28]. EC leads to oxidative stress, a status producing excessive ROS, and a variety of systemic and local inflammatory responses [29]. These adverse effects of EC were clearly demonstrated in the current study, as male and female young subjects, regardless of dietary supplementation, showed increased ROS generation from neutrophil respiratory burst (NRB), activated neutrophil expression, and overproduction of pro-inflammatory cytokines and chemokines, at 4–24 h post EC. Notably, these immunological and inflammatory responses were associated with enhanced muscle cell damage indicated by CK release and pain sensation, and in a delayed fashion typical of EC-induced muscle destruction [1–4].

Recent research indicates that supplementing large doses of antioxidants and/or anti-inflammatory drugs from pharmaceutical sources to suppress EC-induced inflammation can be detrimental, because they may interfere with and inhibit redox signaling pathways that elicit beneficial exercise adaptations [30, 31]. Thus, seeking natural food sources of phytochemicals as dietary supplements with antioxidant and anti-inflammatory properties is highly desirable [32–34].

AVA were first discovered as phytoalexins with antimicrobial effect (inhibiting fungal germination in vivo) in response to infection [35, 36]. Over the past two decades, increasing research findings have revealed AVA’s antioxidant and anti-inflammatory properties in both animal and human studies [12–14]. Direct evidence that AVA can reduce oxidative stress and inflammatory responses were reported by Koenig et al. [13, 14] in which young and older women supplemented with two AVA-enriched cookies daily for 8 weeks decreased ROS generation from NRB and plasma CRP levels after a DR protocol similar to that in the present study. AVA-treated women groups demonstrated lower levels of mononuclear cell NF-κB activation and plasma IL-1β concentration. In the present study, we report several additional lines of evidence that not only substantiated the efficacy of AVA in mitigating inflammatory responses to stressful muscular activity reported in our earlier studies, but also shed some light on the mechanism of AVA protection. First, we confirmed that NRB plays a key role in ROS generation during EC and that chronic consumption of oats containing high AVA content can reduce this source of ROS generated from neutrophils (or monocytes) catalyzed by NADPH oxidase [8, 37, 38] (Fig. 3). We further found that G-CSF might play an important role in facilitating NRB during DR, and AVA was able to attenuate this function (Fig. 4). CSFs are a group of cytokines that are released from injured skeletal muscles that promote the proliferation, differentiation and activation of hematopoietic cells after EC-induced muscle damage [39–41]. Previous research showed that plasma G-CSF levels were significantly elevated several hours after an acute bout of eccentric exercise and reached peak concentrations at 24 to 48 h post exercise [7, 42–45]. At the injury site, these recruited phagocytic cells remove and degrade damaged tissue by engulfing cellular debris (degranulation and phagocytosis) and releasing proteases and ROS [6, 46]. ROS-activated NF-κB pathway amplifies the inflammation signals by expressing pro-inflammatory cytokines such as IL-1β, IL-6 and TNF-α and adhesion molecules to escalate inflammation [47]. Thus, our data showing that AVA-supplemented subjects has lower G-CSF levels in peripheral circulation suggest that this granulocyte-stimulating cytokine might facilitate neutrophils to generate ROS during DR and that by suppressing G-CSF expression AVA may ameliorate inflammatory process.

Second, our present study provided some new data on the effect of oats and AVA on downregulating plasma pro-inflammatory cytokines and chemokines in response to eccentric exercise. IL-6 can be derived from both macrophages and damaged muscle cells [48], and was found to increase its plasma concentration immediately and several hours after DR during both pre- and post-supplementation (Fig. 5), which was also reported in several previous studies [39, 44, 45, 49, 50]. After consuming AVA-enriched oat cookies for 8 weeks, the AVA-supplemented subjects showed an overall reduction of plasma IL-6 level (*P* = 0.082) despite being subjected to the same exercise stress as the control subjects. Although not statistically significant, this trend was consistent with our previous report that dietary AVA supplementation significantly reduced plasma IL-6 levels 24 h after DR in young women [14], and suppressed DR-induced plasma IL-1β and CRP levels in postmenopausal women [13]. We further demonstrated that anti-inflammatory cytokine IL-Ra concentration was elevated in response to DR and that AVA treatment significantly increase plasma IL-1Ra levels during post-supplementation session (Fig. 6). As the IL-1 receptor antagonist (also known as IL-1 inhibitor), IL-1Ra blocks the binding activities of both IL-1α and IL-1β to IL-1R thus attenuating a major source of inflammatory cytokine activation [51].

In addition to pro-inflammatory cytokines, we examined plasma levels of cell adhesion molecules (CAMs) such as VCAM-1 and MCP-1, which are known to facilitate neutrophil and macrophage infiltration into muscle cell to induce inflammatory response following damaging muscular contraction [41, 45, 52]. As a group of transmembrane proteins, CAMs are expressed on the surface of leukocytes and endothelial cells which involve the cell-to-cell interaction and migration from the circulation to the injured tissues [53–57]. We found that both sVCAM-1 and MCP-1 levels in the blood were increased in response to DR, which was consistent with previous reports [44, 45], whereas a significant attenuation of sVCAM-1 and a trend toward decreased MCP-1 were observed during the post-supplementation trials (Fig. 7, 8). Moreover, AVA-supplemented subjects showed lower levels of plasma sVCAM-1 than those consuming plain oats containing no AVA. Based on these findings, we speculate that AVA as well as other antioxidant ingredients in oats are capable of decreasing blood adhesion molecule expression thereby indirectly decreasing the migration and infiltration of monocytes during eccentric exercise [4, 58]. We further speculate that these anti-inflammatory effects of AVA were conferred by the inhibition of NF-κB pathway, as demonstrated by previously reported studies [13, 14, 22]. Clear evidence on the role of NF-κB was derived in a recent study showing that t-butyl-hydroperoxide-stimulated TNF-α and IL-1β expressions in C2C12 muscle cells were downregulated by three major fractions of AVA (A, B and C) [15]. Importantly, protein-ligand docking and molecular dynamics simulation studies revealed that AVA acted as an allosteric inhibitor for IκB kinase (IKKβ), the primary activator of NF-κB pathway. In another recently published study, AVA was found to inhibit TNFα-induced ROS generation, NF-κB activation, and production of IL-1β and IL-6 in C2C12 muscle cells [16]. These data suggest that the reduced plasma inflammatory profiles seen after AVA supplementation may result from attenuated NF-κB activation in skeletal muscle in response to eccentric exercise.

Finally, our present study indicated that chronic oat consumption not only ameliorated plasma inflammatory response to exercise stress, but also mitigated muscle damage as revealed by a lower plasma CK level (Fig. 2). Furthermore, pain sensation by the subjects, most likely related to DR-induced muscle damage, was reduced during post-supplementation trials especially among subjects treated with AVA-enriched diet (Fig. 9). These physiological data support the notion that besides the general benefits of oat consumption derived from various antioxidant and anti-hyperglycemic ingredients, long-term dietary supplementation of AVA may provide specific protection against detrimental impact of damaging muscular exertion.

## Data Availability

The datasets used and/or analyzed during the current study are available from the corresponding author on reasonable request.

## Abbreviations

AVA: Avenanthramides
CAM: Cell adhesion molecule
CK: Creatine kinase
CRP: C-reactive protein
CSF: Colony stimulating factor
DHR: Dihydrorhodamine
DR: Downhill running
EC: Eccentric contraction
G-CSF: Granulocyte colony-stimulating factor
ICAM: Intercellular adhesion molecule
IKK: IκB kinase
IL: Interleukin
IL-1Ra: Interleukin-1 receptor antagonist
MCP: Monocyte chemotactic protein
NF-κB: Nuclear factor-κB
NRB: Neutrophil respiratory burst
ROS: Reactive oxygen species
TNF-α: Tumor necrosis factor-α
sVCAM: Soluble vascular cell adhesion molecule
